# Risk factors for the development of contrast-induced nephropathy in ICU patients

**DOI:** 10.1186/cc13566

**Published:** 2014-03-17

**Authors:** S Pillai, S Alderson, A Manoras, J Papanikitas, D Lewis, S McKechnie

**Affiliations:** 1 Morriston Hospital, Swansea, UK; 2 John Radcliffe Hospital, Oxford, UK

## Introduction

Critically ill patients cared for in ICUs often require radiological investigation using iodinated contrast agents. Contrast- induced nephropathy (CIN) - a form of acute kidney injury (AKI) - is a complication following the use of such contrast. Although CIN has been thoroughly studied in some populations (for example, those undergoing coronary angiography), it has not been investigated in large numbers of ICU patients [1].

## Methods

We conducted a single-centre retrospective review of the electronic patient records of general adult ICU patients who received iodinated contrast over a 3-year period (2009 to 2011). Our review identified: patient demographics; ICU admission details (specialty, time of admission, elective/emergency admission); physiological status pre scan (evidence of shock, eGFR, urine output); volume of iodinated contrast; and evidence of CIN or AKI post scan (assessed using CIN and KDIGO criteria). Patients were excluded if they had pre-scan renal replacement therapy. Analyses investigated the risk factors for CIN or AKI using chi-squared tests for categorical variables and nonparametric tests for continuous variables (as no continuous variable was normally distributed, even with log transformation).

## Results

In total, 479 scans involving 331 patients were included. A total of 266 (56%) scans were associated with CIN. Significant risk factors for the development of CIN included male gender (*P *= 0.02), reduced prescan GFR (*P *< 0.001), and decreasing time from admission to scan (*P *= 0.03). Ninety-five (20%) scans were associated with the development of AKI. Significant risk factors for AKI again included male gender (*P *= 0.009), reduced pre-scan eGFR (*P *< 0.001), and decreasing time from admission to scan (*P *= 0.003), but also included emergency admission to ICU *(P *= 0.03), pre-scan shock *(P <*0.001), and pre-scan oliguria (*P *< 0.001).

## Conclusion

Male gender, reduced pre-scan eGFR and decreasing time from admission to scan are risk factors for the development of both CIN and AKI. The association with time from admission to scan may reflect inadequate patient optimisation prior to contrast administration; this hypothesis is supported by the risk factors significantly associated with AKI alone (emergency admission to ICU and pre-scan shock). Given the adverse clinical outcomes associated with the development of CIN/AKI, these findings necessitate a review of current practice.

## References

[B1] Lameire Contrast-induced acute kidney injury and renal support for acute kidney injury: a KDIGO summary (Part 2) Crit Care 2013 17 205 10.1186/cc11455 23394215 PMC4056805

